# New perspectives in the study of the Earth’s magnetic field and climate connection: The use of transfer entropy

**DOI:** 10.1371/journal.pone.0207270

**Published:** 2018-11-15

**Authors:** S. A. Campuzano, A. De Santis, F. J. Pavón-Carrasco, M. L. Osete, E. Qamili

**Affiliations:** 1 Istituto Nazionale di Geofisica e Vulcanologia (INGV), Roma, Italy; 2 Dipartimento di Ingegneria e Geologia, Università degli Studi “G. D’Annunzio”, Chieti, Italy; 3 Dpto. de Física de la Tierra y Astrofísica, Universidad Complutense de Madrid (UCM), Madrid, Spain; 4 Instituto de Geociencias (IGEO) CSIC, UCM, Ciudad Universitaria, Madrid, Spain; 5 Serco SpA, Frascati, Rome, Italy; Universidade Estadual de Maringa, BRAZIL

## Abstract

The debated question on the possible relation between the Earth’s magnetic field and climate has been usually focused on direct correlations between different time series representing both systems. However, the physical mechanism able to potentially explain this connection is still an open issue. Finding hints about how this connection could work would suppose an important advance in the search of an adequate physical mechanism. Here, we propose an innovative information-theoretic tool, i.e. the transfer entropy, as a good candidate for this scope because is able to determine, not simply the possible existence of a connection, but even the direction in which the link is produced. We have applied this new methodology to two real time series, the South Atlantic Anomaly (SAA) area extent at the Earth’s surface (representing the geomagnetic field system) and the Global Sea Level (GSL) rise (for the climate system) for the last 300 years, to measure the possible information flow and sense between them. This connection was previously suggested considering only the long-term trend while now we study this possibility also in shorter scales. The new results seem to support this hypothesis, with more information transferred from the SAA to the GSL time series, with about 90% of confidence level. This result provides new clues on the existence of a link between the geomagnetic field and the Earth’s climate in the past and on the physical mechanism involved because, thanks to the application of the transfer entropy, we have determined that the sense of the connection seems to go from the system that produces geomagnetic field to the climate system. Of course, the connection does not mean that the geomagnetic field is fully responsible for the climate changes, rather that it is an important driving component to the variations of the climate.

## Introduction

The possible relationship between the Earth’s climate and geomagnetic field has been highly debated in the last fifty years (e.g. [1, 2, 3, 4, 5, 6, 7, 8, 9]) but it is still an open question. The first serious proposals, that quantify this possible link, were given by Wollin et al. [1], who pointed out that low geomagnetic intensities are generally associated with warm climate periods (similar to the current situation), and by Bucha [10], who suggested that drifts of geomagnetic poles could have been responsible for displacements of a large low-pressure region of the Earth’s atmosphere associated with an increase of cyclonic activity and sudden climate changes [11].

Throughout the last few decades, other mechanisms that could explain the geomagnetic field-climate relation have been proposed (e.g. [3, 4, 6, 8]). The most plausible at long-time scale is related to the rate of galactic cosmic rays coming to the Earth’s surface. This flux of galactic cosmic rays is modulated by the intensity of both Sun and the Earth’s magnetic fields that act as a protective shield. High values of the solar (and Earth’s) magnetic field intensity reinforce the shield and then a low density of galactic cosmic rays coming to the Solar System (and in turn to Earth) is expected [12]. Entering the atmosphere, the cosmic rays could play an important role in cloud formation [13, 14] and, in this way, the geomagnetic field would be involved in climate processes. That is, a decreasing in the geomagnetic field intensity would allow a higher entrance of galactic cosmic rays to the Earth that could enhance the formation of low-lying clouds [15, 16, 17] or increase the global cloud cover leading to tropospheric cooling [3]. This mechanism was invoked to explain the possible relation between the intensity of Earth’s magnetic field and climate on glacial-interglacial timescales, since dipole moment lows (related to geomagnetic excursions) seem to occur shortly before the onset of relatively cold intervals [6, 8]. This suggests a connection between low geomagnetic intensity and climatic cooling. However, such connection could be circumstantial, as pointed out by these authors, since the variations in geomagnetic field intensity may, in fact, be linked to variations in Earth’s orbital parameters [6], which are considered the main climate-controlling factors in the Pleistocene [18]. Dergachev et al. [19] also studied the relation between short-term geomagnetic variability (jerks) and climate change, as well as the accelerated drift of the north magnetic pole and surface temperature variations. They also propose as more probable mechanism, a relation between the entrance of cosmic rays and formation of clouds.

On the other hand, Gallet et al. [4] compared the advance and retreat of the Alpine Glaciers during the last three millennia with increases and decreases of the geomagnetic field intensity in Paris estimated from archeomagnetic data (paleomagnetic data from heated archaeological artefacts). A later work with a more complete paleomagnetic intensity database corroborated a similar connection at European continental scale [20]. The results of these studies suggest a possible link between centennial-scale cooling episodes and enhanced geomagnetic intensity, the opposite to the galactic cosmic rays mechanism [3, 6, 8, 16, 17] but in agreement with the first links established in the 70’s [1, 10, 11].

Other studies point out other possible mechanisms that explain this connection, such as the experimental result of Pazur and Winklhofer [21]. They focus on the effect of the geomagnetic intensity on CO_2_ solubility in the ocean. They observed that low values of geomagnetic field intensity reduce the CO_2_ solubility in the ocean, displacing more CO_2_ to the atmosphere and increasing the temperature.

For shorter time scales, i.e. last 300 years, De Santis et al. [22, 23] observed a similar temporal trend between the growing South Atlantic Anomaly (SAA) area extent on the Earth’s surface and the Global Sea Level (GSL) rise. The SAA is one of the most outstanding features of the geomagnetic field. It is a large geomagnetic anomaly, presently covering a large area over the Western coast of Africa, the South Atlantic Ocean, the major part of South America and the South-eastern Pacific Ocean, which reaches lower values of intensity than expected at those geomagnetic latitudes. Several studies [24, 25, 26, 27, 28] point out that this anomaly is the response on the Earth’s surface of reversed flux patches located at the terrestrial CMB (core-mantle boundary). De Santis et al. [22] proposed three mechanisms to explain this possible link based on the entrance of charged particles from space, the possible reduction of the ozone layer in the upper stratosphere over the South Atlantic region and/or a common internal cause shared between both SAA and GSL time variations.

All these works and physical mechanisms proposed lead to the deduction that the possible link between the Earth’s climate and the geomagnetic field is far from being demonstrated and understood.

In this work, we propose to study, for the first time, the possible causal information link between two previously studied real time series by means of an innovative statistical tool for non-linear dynamic studies that measures the information flux and the sense of this flux: Transfer Entropy (TE) [29]. This measure has been used in other scientific fields for the last decades, for example in the climatic context [30, 31] or in the geomagnetic activity studies [32]. We will apply it on the SAA surface extent and GSL rise for the last 300 years following De Santis et al. [22] but on shorter scales. We choose these two time series because are important in the frame of the natural hazards. The present strong decrease of the main geomagnetic dipole field could eventually indicate a reversal (e.g. [33, 34]). As well, it plays a main role in screening most of the solar and galactic radiation from space, otherwise penetrating in a larger quantity into the atmosphere and causing possible health and environmental damages. In addition, understanding whether the present increasing trend of the GSL is continuing or not in the close future is vital because of the possible increase of new lands coverage by sea.

The present paper is structured as follows: in the first section, we expose the chosen time series to carry out this analysis. Then, we explain the details on the main methodologies applied in this work. Finally, in the discussion and conclusions we summarize the outcomes reached and their possible future implications.

## Data

We analyze two time series: a) the SAA area extent at the Earth’s surface given by historical geomagnetic field models (GUFM1 model, [35]; and the later modifications [27, 36]), and b) the GSL reconstruction for the last 300 years [37]. Both time series are detailed below.

**Fig 1:**
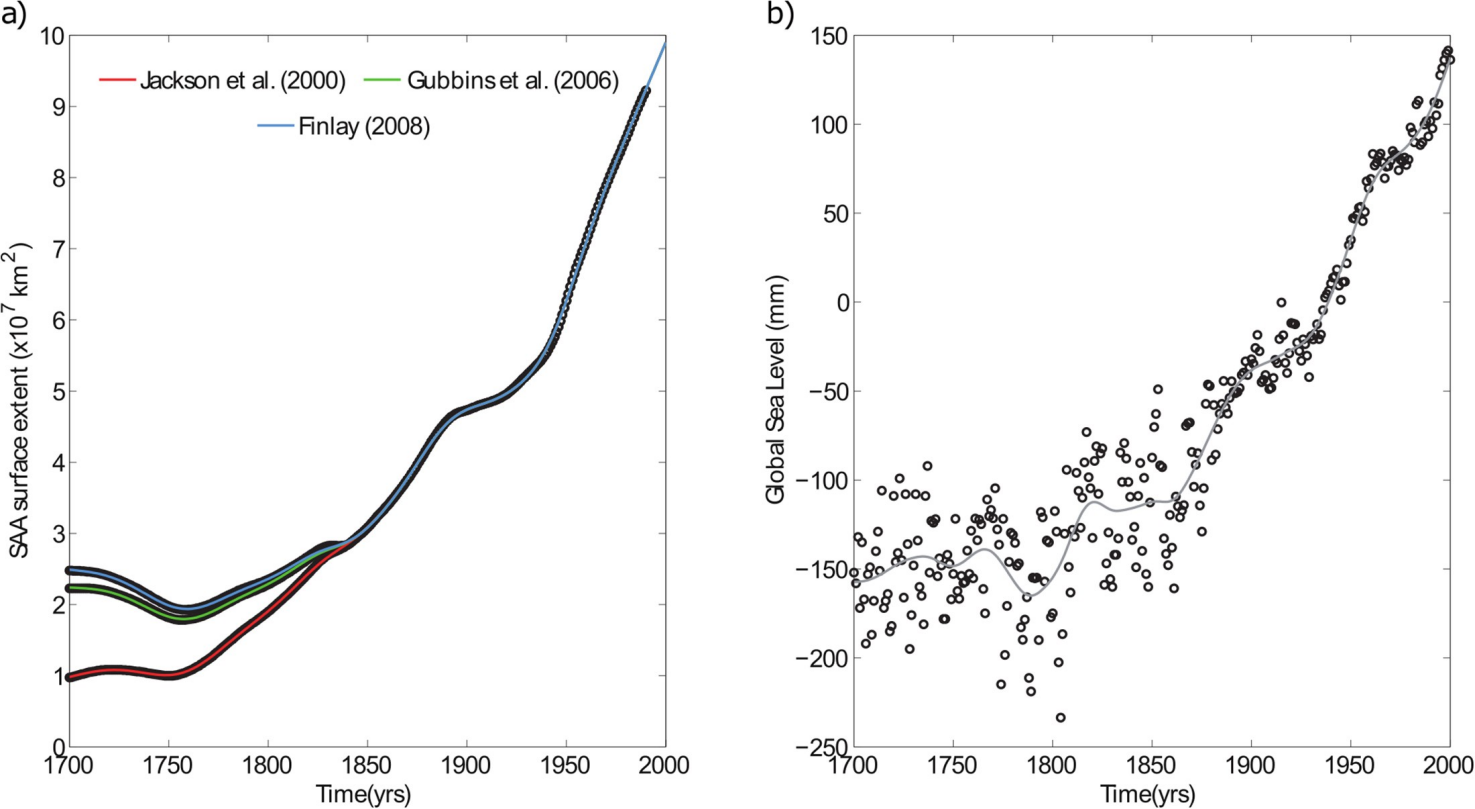
Time series evolution. Evolution of a) the SAA area extent (within the 32000 nT isoline of the geomagnetic field) on the Earth’s surface in km^2^ from three global geomagnetic field models [27, 35, 36] and b) GSL rise in mm, for the last 300 years (1700–2000). The lines represent the fits by using penalized cubic splines: (red, green, blue) SAA derived from Jackson et al. [35], Gubbins et al. [27] and Finlay [36], respectively, and (gray) GSL.

The SAA surface extent could be defined, in practice, by the area below a given intensity contour line at the Earth’s surface (here we selected the contour line of 32000 nT following De Santis et al. [22]). The SAA surface extent has been computed from the three mentioned historical geomagnetic field models covering the last 400 years. The difference between these models lies in the method used to estimate the first Gauss coefficient (g_1_^0^) prior to 1840 AD, due to the lack of instrumental intensity data before that year. Jackson et al. [35] extrapolated linearly the value of this coefficient backwards from 1840 and they assumed a constant rate of temporal evolution of 15 nT/yr, which corresponds to the average time rate of g_1_^0^ from 1850 to 1990. Gubbins et al. [27] modified the g_1_^0^ by using the intensity paleomagnetic database [38] for the period from 1590 to 1840 to obtain a more realistic value of this coefficient. More recently, Finlay [36], using the same paleomagnetic database, applied different statistic approaches to fix again the coefficient g_1_^0^ providing no rate of change for that coefficient from 1590 to 1840. Consequently, the estimations of the SAA surface extent obtained by these models differ slightly for times prior to 1840, but agree for the most recent period (see Fig 1a).

For the Global mean Sea Level (GSL), we use a reconstruction since 1700 based on the longest available tide-gauge records [37] (http://www.psmsl.org/products/reconstructions/jevrejevaetal2008.php), where the effects of vertical land movement induced by the glacial isostatic adjustment of the solid Earth have been removed. Jevrejeva et al. [37] extended the record backwards from 1850 using three of the longest (though discontinuous) tide-gauge records available, being the error of the reconstruction higher in this epoch (Fig 1b).

We have smoothed both the SAA and the GSL series by using penalized cubic splines in order to avoid future mathematical artefacts resulting from the differences in the reconstruction prior and after 1850. For both records, the fitting was carried out using knot points every 5 years from 1700 to 2000 and a spline damping parameter of 10 yr^4^/km^4^ and 10 yr^4^/mm^2^ for the SAA and GSL time series, respectively. These optimal values were estimated according to the root mean square (rms) error (see Fig A in S1 File).

**Fig 2:**
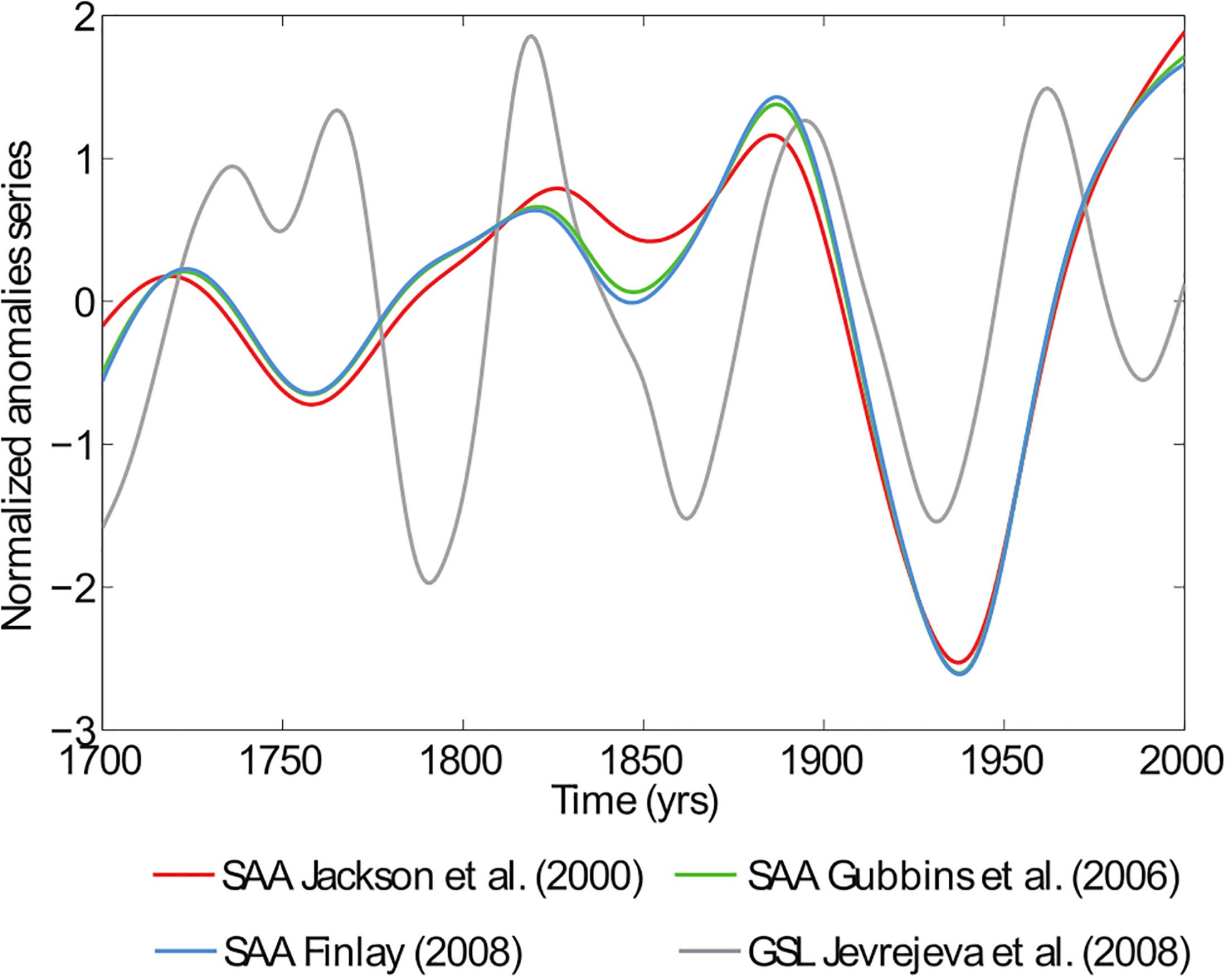
Evolution of the time series anomalies. Red, green, blue lines correspond to SAA anomalies derived from Jackson et al. [35], Gubbins et al. [27] and Finlay [36], respectively. Grey line represents the GSL anomalies. See text for further details. Both time series have been normalized to zero mean and unit variance.

In general, the Transfer Entropy (TE) is applied on stationary time series [39]. However, as evident from Fig 1, both SAA and GSL series cannot reasonably be assumed as stationary, being both curves almost monotonically increasing. For this reason, we will apply the TE to the anomaly time series after removing the best-fit long-period trend (see Fig 2). In our case, we choose the simplest polynomial function that accounts for the time evolution of the series: a second order polynomial, which seems the best compromise to remove a reasonable trend and not to completely destroy some similar short-period fluctuations in both series. A positive/negative anomaly would mean that the SAA area extent or GSL rise grow more/lesser than expected.

## Methods

TE is an information theoretic measure introduced by Schreiber [29] as a generalization of the mutual information [40]. While the mutual information contains neither dynamics nor directional information, the TE takes into account the dynamics of information transport between two systems. This allows quantifying both the exchange of information to the predominant sense of this flow.

The foundations of the TE are to be found in the basic works of the theory of information [41]. The Shannon entropy is given by:

$$H_{I}=-\sum_{i}p(i)l{og}_{2}p(i),$$ (1)

where *i* represents the states that the process *I* can assume and *p(i)* the probability distribution which they follow. This quantity measures the average amount of information needed to encode a process optimally.

From finite-order Markov processes, Schreiber [29] introduced a measure to quantify information transfer between two different time series, based on appropriately conditioned transition probabilities instead of static probabilities. Assuming that the system under study can be approximated by a stationary Markov process of order *k*, the transition probabilities describing the evolution of the system are *p*(*i*_*n*+1_|*i*_*n*_, …, *i*_*n*−*k*+1_). If two processes *I* and *J* are independent, then the generalized Markov property

$$p(i_{n+1}|i_{n},\ldots ,i_{n-k+1})=p(i_{n+1}|i_{n}^{\left(k\right)},j_{n}^{\left(l\right)}),$$ (2)

holds, where $i_{n}^{\left(k\right)}=(i_{n},\ldots ,i_{n-k+1}),\;j_{n}^{\left(l\right)}=(j_{n},\ldots ,j_{n-l+1})$ and *l* indicates the number of conditioning states for process *J*.

Schreiber [29] proposed, using the Kullback entropy for conditional probabilities [42, 43], to measure the incorrectness of assuming the generalized Markov property (Eq [2]), i.e. *I* and *J* are independent, which results in:

$${TE}_{J\to I}=\sum p(i_{n+1},i_{n}^{\left(k\right)},j_{n}^{\left(l\right)})log\frac{p(i_{n+1},i_{n}^{\left(k\right)},j_{n}^{\left(l\right)})p(i_{n}^{\left(k\right)})}{p(i_{n}^{\left(k\right)},j_{n}^{\left(l\right)})p(i_{n+1},i_{n}^{\left(k\right)})},$$ (3)

denoted as transfer entropy (a schematic representation of the TE can be found in Fig B in S1 File). The TE can be understood as the excess amount of information that must be used to encode the state of a process by erroneously assuming that the actual transition probability distribution function is $p(i_{n+1}|i_{n}^{\left(k\right)})$, instead of $p(i_{n+1}|i_{n}^{\left(k\right)},j_{n}^{\left(l\right)})$.

The TE computation on real time series has some shortcomings and limitations that must be addressed in the best possible way: 1) the choice of the strategy followed to calculate the TE: discretization method and optimal parameters. The results depend on the different parameters used and it is important to check that we find approximately invariant results with different sets of them. 2) The finite sample size of the real time series: it is always necessary check that the number of data is enough to apply the TE. By examining the log posterior probability for the optimal number of bins *S* used to discretize the time series, it is possible to verify whether one possesses sufficient data, and it is the method used in this work. 3) The interpretation of the TE results: Smirnov [44] pointed out the inability of the TE to differentiate indirect influences from direct influences. In general, the most widely used interpretation of the TE is to consider that, if it exists, this means that there is an information flow or transfer between the two time series analyzed (*I*, *J*). James et al. [45] found that transfer-like entropies could both overestimate information flow and underestimate influence. They proposed a new interpretation of the transfer entropy as a measure of the reduction in uncertainty about one time series given another, instead of as information flow or transfer, which is understood as the existence of information that is currently in *I* is caused solely by *J*’s past.

There are different strategies to calculate the TE from the analysis of real data. Here, we use the method based on the discretization of the time series, which was explained in detail by Sandoval Jr [46]. This method consists in dividing the data in a number of bins *S*, by assigning a numeric symbol to each bin from 1 to *S*. Each symbol corresponds to a range of values of data series, which are replaced by the symbols assigned (from 1 to *S*).

Obviously, the calculation of TE will depend on the specific partition chosen *S*. In order to obtain the optimal number of bins *S*, we consider the approach proposed by Knut [47], where *S* is given by the maximization of the posterior probability *p*(*S*|*N*, *n*_*k*_). Given a uniform bin-width histogram for a statistical data set of *N* samples, the posterior probability *p*(*S* ∣ *N*, *n*_*k*_) is given by:

$$p(S|N,n_{k})\propto {\left(\frac{S}{V}\right)}^{N}\frac{\Gamma (S/2)}{\Gamma {\left(1/2\right)}^{S}}\frac{\prod_{k}^{}\Gamma (n_{k}+1/2)}{\Gamma (N+S/2)},$$ (4)

where *n*_*k*_ is the number of samples in the *k*^*th*^ bin, *V* is the data range length, and Γ is the Gamma function. In optimization problems, it is common to maximize the logarithm of the Eq [4] [47], also because from the behaviour of the logarithm one can study if the chosen time series are sufficiently long to be analyzed with a tool like the TE [48]. For this reason, we maximize the logarithm of the posterior probability to, firstly, determine if the chosen time series are long enough and then, estimate the optimal number of bins.

Once we have checked that the number of data is enough and estimated the optimal number of bins *S*, we discretize the time series as we explained above, and compute directly the TE from the Eq [3] given by Schreiber [29], with $i_{n}^{\left(k\right)}$ and $j_{n}^{\left(l\right)}$ representing both involved series. The choice of the embedding dimension *k* and *l* is a key point in the computation of the TE. If the dimension is too low, the information contained in the past time (or memory) of the series *I* might be assigned to come from *J*. In order to avoid this, we must get that the series *I* is independent from itself with a delay *k*. Therefore, we base the selection of this parameter on the determination of the mutual information between the time series *I* and itself with a delay *k* [49]:

$$M_{II_{k}}(k)=\sum_{i,i_{k}}^{}p(i,i_{k})log\frac{p(i,i_{k})}{p(i)p(i_{k})},\;$$ (5)

being *I*_*k*_ the time series *I* with delay *k*. The value of *k* associated with the first local minimum reported in the Eq [5] is considered the optimal embedding dimension.

For the dimension of the embedding *l* of the *J* series, it is usually considered *l* = 1 or *l* = *k* [29, 39]. In a conservative approach we consider *l* = 1. To calculate the different probabilities of the Eq [3] we simply count the number of times that a symbol or sequence of symbols appears in our time series.

Due to finite size of the time series and the reduced data number, the establishment of a threshold at which the result can be considered significant is essential. In order to establish the statistical significance of our results we calculate the TE with the data points of the *J* series, which represents the source of the presumed information flow, shuffled randomly [39, 50]. The objective of this procedure is to destroy all potential relations between the two series, *I* and *J*, and hence the observed TE should be zero. In finite time series this value rarely is zero due to the finite sample effects, and we obtain the threshold value of TE above which is significant. Practically, we create 1000 surrogate time series of *J* by using the Iterated Amplitude Adjusted Fourier Transform technique (IAAFT) [51, 52, 53]. This procedure assures that the surrogate time series have the same mean, variance, autocorrelation function and therefore, power spectrum as the original series but destroys the non-linear relations and, therefore, the information actually significant transferred from *J* to *I* series. To consider the original TE significant we consider the 5% null hypothesis being the null hypothesis that the transfer entropy between the two original time series is not significant. Whether the 95% of the new TEs values, calculated from surrogate series *J*, are lesser than the original one, then we consider the original TE significant.

## Results and discussion

**Fig 3:**
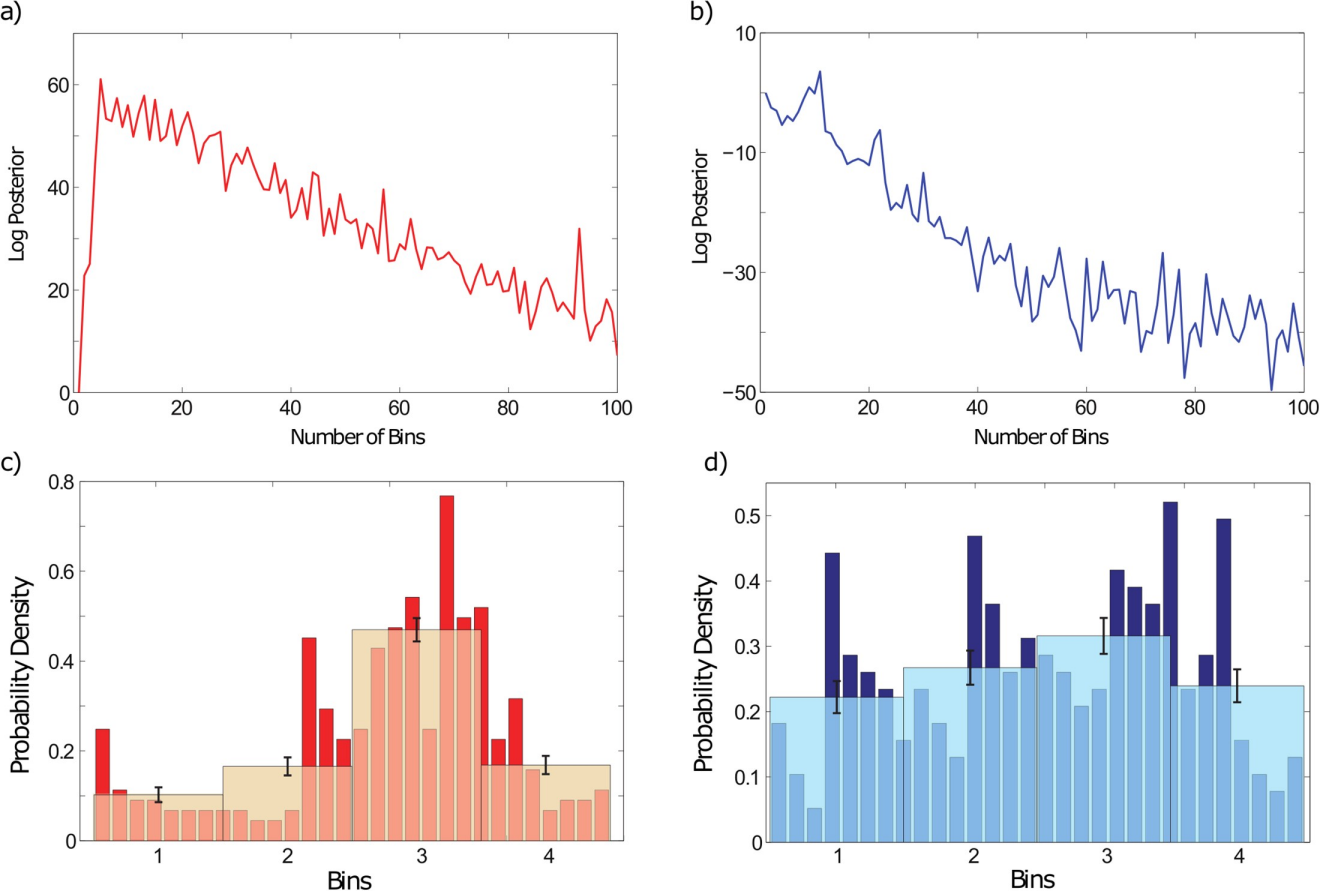
Evaluation of the length of time series and optimal number of bins. Log posterior curves in function on the number of bins *S*: a) for the SAA anomalies computed from Jackson et al. [35] and b) for the GSL anomalies. The subplots c) and d) represent, in orange and cyan respectively, the chosen discretization (*S* = 4) taking into account the results given in a) and b), as well as the main characteristics of the probability density of both systems (see red and blue bars in c) and d) plots). The error bars indicate the standard deviation of the bin heights.

The analysis of the logarithm of Eq [4] (log posterior) in function on the number of bins provides useful information: a) both time series are long enough to apply the TE and b) the selection of the optimal number of bins *S* according to the maximum in the log posterior function (see Fig 3a and 3b). The log posterior of SAA anomalies (Fig 3a) increases sharply according to the number of bins considered, reaching a peak (corresponding to the optimal number of bins *S* = 5) and then decreasing. Respect to the GSL anomalies series (Fig 3b), the log posterior also decreases gradually but the maximum is not so clear. These behaviors indicate a sufficient amount of data to develop this analysis with the TE, but finite sample effects could be important. Due to the lack of an obvious peak in the GSL anomalies series, we establish an agreement between the log posterior curve and the main characteristics of the histogram of the time series. In view of Fig 3d, we consider that with *S* = 4 we have captured the main information of this series (see also Fig C in S1 File). Finally, in order to avoid a future bias in the computation of the TE, we choose the same number of bins *S* for both time series i.e., equal to 4 (see Table 1 and Fig C in S1 File) due to larger bin sizes (smaller *S*) are usually favored in the literature because show the differences more sharply [46].

**Table 1:** Optimal parameters.

OPTIMIZATION PARAMETERS				
	SAA surface extent			GSL
	*Jackson et al*. [2000]	*Gubbins et al*.[2006]	*Finlay* [2008]	
**S** **k**	4 26	4 26	4 26	4 13

Selection of the optimal number of bins *S* and the embedding dimension *k* for SAA and GSL anomalies.

As indicated in the methodology, the selection of the embedding dimension *k* for both series was carried out using the mutual information given by Eq [5]. Results are plotted in Figs Da and Db and contained in the Table Ab in the S1 File. For the GSL anomaly series the optimal dimension was obtained for *k*_*GSL*_ = 13, while different values were obtained for the 3 SAA anomalies series (24 for the SAA anomalies series of Jackson et al. [35]; and 26 for the other two series). Nevertheless, since different embedding dimensions can generate TE bias [54], we have fixed the dimension *k*_*SAA*_ in 26 for all the 3 SAA anomalies series because a slight over-embedding does not compromise the detection of significant TE [55]. To corroborate the different value of dimension *k* for GSL and SAA series, we have also calculated the autocorrelation function since the simplest estimate of an optimal *k* is the first zero of the autocorrelation function [56, 57]. The problem is that these estimates generally yield too large *k* values for stochastic dynamical systems [58]. In fact, the first minimum reported for the SAA anomalies is given in *k*_*SAA*_ = 29 and for the GSL anomalies in *k*_*GSL*_ = 17 (Figs Dc and Dd in S1 File). As provided by the mutual information (*k*_*SAA*_ = 26 and *k*_*GSL*_ = 13), the autocorrelation functions also indicate a lower memory for the GSL series than the three SAA series.

In order to evaluate how the selection of these parameters (*S*, *k*) affects the results, we have performed several tests using different sets of them. The results are detailed in the S1 File along with the Tables A and B. In addition, we have performed different tests to study the impact of a) the use of a different detrending approach to define the anomalies (Fig E and Table C in S1 File) and b) the use of an unsmooth GSL time series (Fig F and Table D in S1 File). Detailed information about these tests could be also found in the S1 File. We find that these changes can slightly affect the statistical significance of our results but not the sense of the information flow between the two time series.

**Fig 4:**
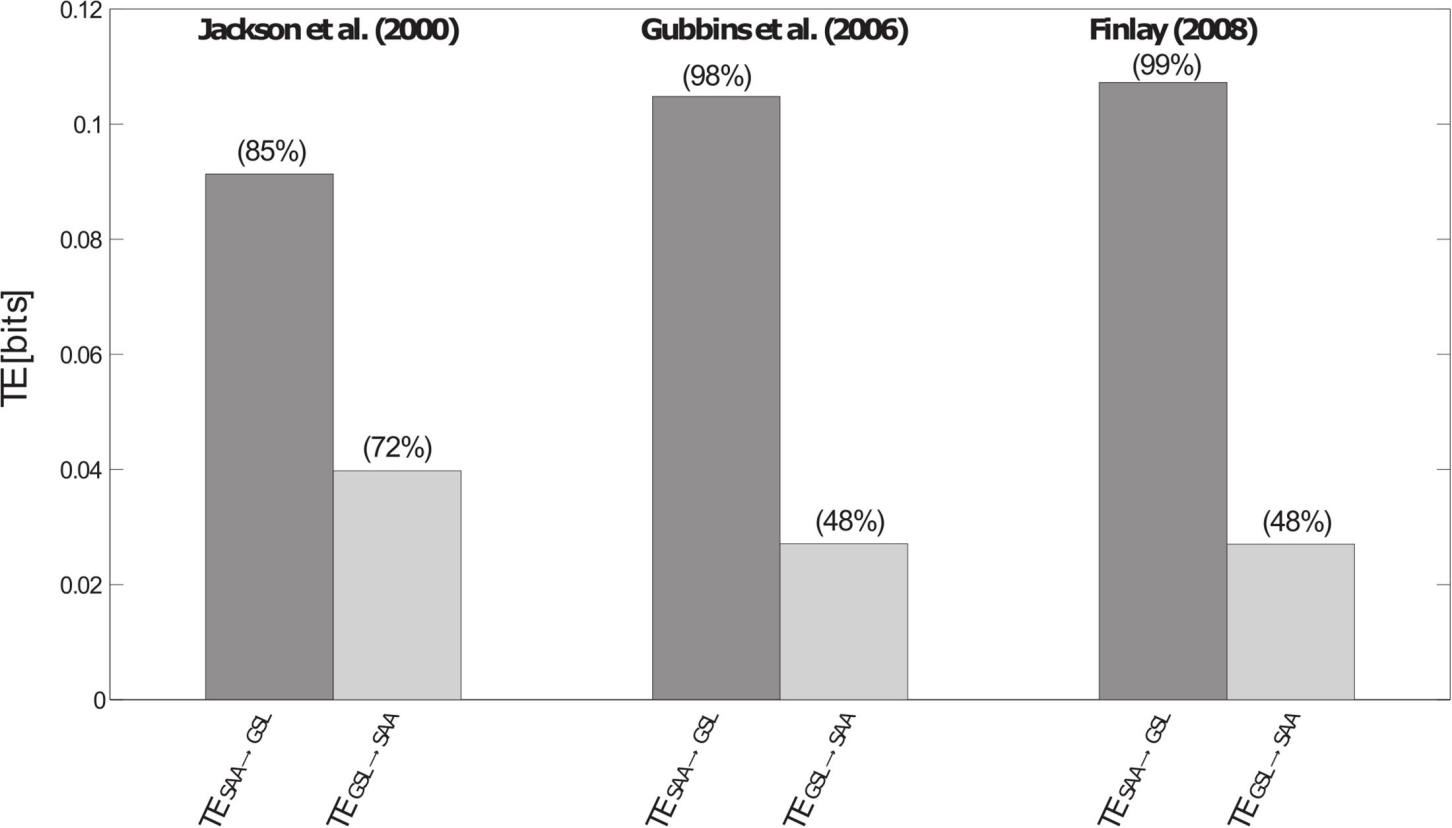
Results of transfer entropy analysis. Transfer entropy by measuring the information flow from SAA to GSL anomalies and from GSL to SAA anomalies, by using the three historical models for the geomagnetic field to compute the SAA surface extent. In brackets, the significant level indicates the percentage of TEs calculated from surrogate series that are lesser than the original TE.

**Fig 5:**
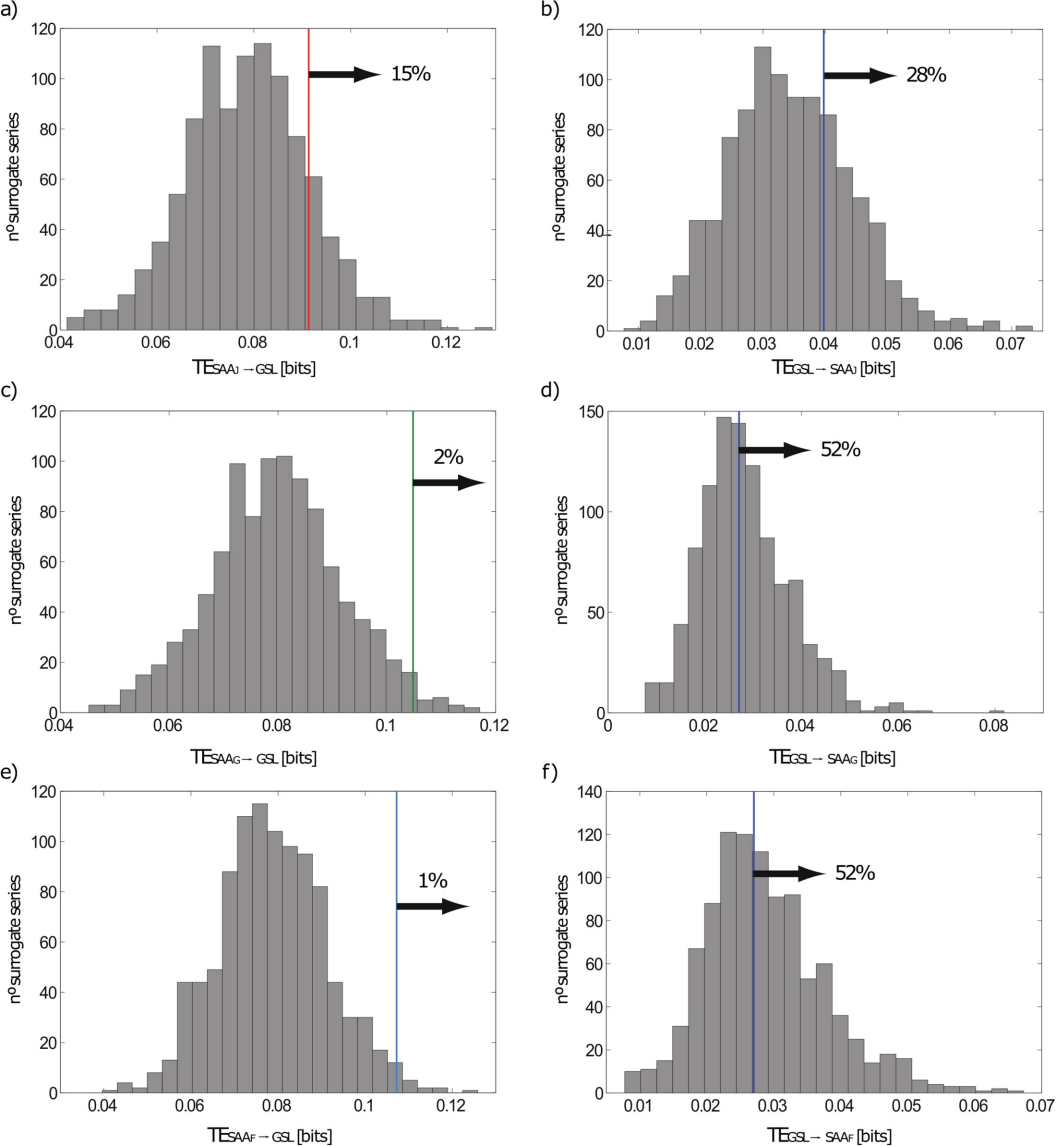
Statistical significance of the transfer entropy results. Transfer entropy calculated from surrogate series a), c) and e) of SAA anomalies from Jackson et al. [35] (SAA_J_), Gubbins et al. [27] (SAA_G_) and Finlay [36] (SAA_F_) respectively and b), d) and f) GSL anomalies. The results show that the statistical significance is higher when the sense of the information goes from SAA to GSL anomalies, also registering greater values of the TE.

For the chosen parameters, the TE results (Eq [3]) are given in Table 2 and Figs 4 and 5. As it can be observed, there is a significant information flow from SAA to GSL anomalies by considering the 5% null hypothesis when the most recent geomagnetic field models given by Gubbins et al. [27] and Finlay [36] are used. Anyway, the significant levels calculated following the IAATF approach are clarifying, with percentages around the 90% in all cases for the TE from SAA to GSL anomalies. This outcome indicates that the SAA anomalies add great predictability to the GSL anomalies by suggesting interactions between the two time series of anomalies at a time scale lower or equal to two consecutive data, i.e. one year. However, more investigations must be carried out about the time delay that needs the influence to propagate between both series (e.g. [32, 30, 59]).

**Table 2:** Results of transfer entropy analysis.

	*Jackson et al*. [2000]	*Gubbins et al*. [2006]	*Finlay* [2008]
TE_SAA→GSL_ [bits]	0.091 (85%)	0.10 (98%)	0.11 (99%)
TE_GSL→SAA_ [bits]	0.040 (72%)	0.027 (48%)	0.027 (48%)

Transfer entropy and statistical significance (in brackets) from SAA to GSL anomalies and from GSL to SAA anomalies, with the optimal parameters (*S* and *k*) reported in the Table 1, and *l* = 1.

In view of these results, it would be expected that a future SAA anomaly taking into account our selected trend generates a GSL anomaly with a time lag of one year or less. Several physical mechanisms are proposed to explain this possible coupling [22]. The first of them is that an increase of the SAA area facilitates the entrance of charged particles from space. If the SAA area extent grows more than it is expected (positive anomaly), then this entrance is favored. As a result we have a warmer atmosphere, which, in turn, implies a consequent melting of major ice caps (Antarctica and Greenland) that finally would cause a greater increasing of the global sea level (positive anomaly). Recent works (e.g. [31, 60, 61, 62, 63]) have found interesting correlations between solar and galactic cosmic rays periodic variations and climatic (such as temperature and rainfall) variations in the region where the SAA is located. The entrance of galactic cosmic rays at the atmosphere depends both solar and Earth’s magnetic fields, hence these correlations could also be influenced by a factor depending on the low geomagnetic intensity due to the SAA presence in the region and its continuous increasing for the last centuries.

Another mechanism proposed is that a possible reduction of the ozone layer in the upper stratosphere over the South Atlantic region can modify the radiative flux at the top of the atmosphere and hence can cause changes in the weather and climate patterns, including cloud coverage. Solanki et al. [64] propose a similar mechanism to explain relation between solar activity and climate based on the fact that the variations in solar activity during an 11-year cycle are more intense at shorter wavelengths, which include UV radiation. The variations in UV radiation modify the concentrations of ozone and lead to changes in the atmospheric circulation dynamics.

As we can observe, these two mechanisms relate the solar activity, the galactic cosmic rays production and the geomagnetic field with the Earth’s climate, by suggesting that all of them can work together and be needed to completely explain the found outcomes.

Finally, an internal mechanism was presented by which a convective dynamism in the outer core could cause a variation of the magnetic field and an elastic deformation at the Earth’s surface [65].

In the analyzed case study, we have shown that the sense of the information goes from SAA to GSL time series (Fig 5). This would discard any physical mechanism in which the climate controls the geomagnetic field and support the mechanisms caused by the presence of the SAA.

## Conclusions

We have applied for the first time a recent statistical tool, transfer entropy, to shed light on the question of a possible link between the Earth’s magnetic field and climate and provide new perspectives in its future analysis. In this work, we have analyzed two real time series with an analogous evolution for the last 300 years, the South Atlantic Anomaly area extent on the Earth’s surface and the Global Sea Level rise. We have analyzed the anomalies of both time series, after removing the long term trend. The results seem to support the existence of an information flow between SAA and GSL anomalies, with larger information transferred from SAA to GSL and a confidence level about 90%. The found connection does not mean that the geomagnetic field is fully responsible of the climate changes, rather that it is an important driving component to the variations of the climate. This result is especially relevant because could help to find a physical mechanism able to explain this connection by discarding those in which the climate controls the geomagnetic field and supporting the mechanisms associated to the geomagnetic field.

Although this work seems to provide a favorable argument to this link, future investigations are needed to completely exploit this issue, for example to check other time series at longer timescales.

## Supporting information

S1 File Detailed description of the supplementary material. Complementary figures and tables. Results from different tests that support the main outcomes described in the main text and some case studies that help to better understand the TE results.
(DOCX)
