# Supplementary material for: New perspectives in the study of the Earth’s magnetic field and climate connection: The use of transfer entropy
Source: PLoS One. 2018 Nov 15;13(11):e0207270. doi: 10.1371/journal.pone.0207270 (PMC6237378; doi:10.1371/journal.pone.0207270)
Supplement: S1 File — Complementary figures and tables. Results from different tests that support the main outcomes described in the main text and some case studies that help to better understand the TE results. (DOCX) [file pone.0207270.s001.docx]

Supplementary Material

New perspectives in the study of the Earth’s magnetic field and climate connection: the use of transfer entropy

S. A. Campuzano*, A. De Santis, F. J. Pavón-Carrasco, M. L. Osete, E. Qamili

* Corresponding Author

E-mail: [saioa.arquerocampuzano@ingv.it](mailto:saioa.arquerocampuzano@ingv.it) (SAC)

# Supplementary File

The supporting information includes figures and tables that complement the analysis carried out along the paper. Fig A represents the root mean square (rms) error in function on different damping parameters (*λ*), used to determinate its optimal value according to the trade-off curves. Fig B is a schematic representation to better understand how works the transfer entropy from a system *J* (SAA anomalies) to other system *I* (GSL anomalies). Fig C shows the discretized time series used to estimate the TE, compared to the original time series of anomalies. Fig D represents the results given by the mutual information and the autocorrelation function to obtain the optimal embedding parameters (*k*_SAA_ and *k*_GSL_). Figs E and F and Tables A-D shows the results of different tests carried out to study the impact on the final results of: a) the use of different sets of optimal parameters (number of bins, *S*, and embedding parameters, *k*) (Tables A and B), b) the use of different detrending approaches (Fig E and Table C) and c) the use of unsmooth GSL time series (Fig F and Table D).

1. Test 1: Use of different sets of optimal parameters (*S*, *k*)

Table B contains the results of TE according to the sets of parameters given in Table A and reflects the results of the tests carried out changing both number of bins (*S*) and embedding parameters (*k* or *l*). We consider different cases for each information flow sense. From SAA to GSL (the other sense, from GSL to SAA, is analogous) we have checked 3 different values for the memory of the SAA time series (*l*) using the optimal embedding parameter for the GSL series (*k*) as follows: a) *l* = 1, b) *l* = *k*, c) *l* equal to the optimal embedding parameter for the SAA series. In order to see how the number of bins affects these results, we have repeated the analysis using the different *S* values contained in Table A.

The results show that the TE increases when more information is available in the system. However, the statistical significance decreases due to the finite sample effect (e.g. [1]). In any case, the best results, according to the obtained significance, are provided by the optimal value of *S* = 4 and *l* = 1. Moreover, regardless of the selection of the number of bins and the embedding dimension, the predominant sense of the TE is from SAA to GSL anomalies. This reinforces the result reached.

Finally we would like to point out what happens when an incorrect number of bins is chosen (see Table Bd). In this case, the optimal value of the GSL anomalies series is badly conditioned (see Fig 3b) registering spurious local maxima. If we consider the highest maximum (*S*=11) as the optimal *S* value, we are overestimating the degrees of freedom of the time series, increasing the amount of information available in GSL anomalies and enhancing the TE from GSL to SAA anomalies. However, the statistical significance is much lesser than in the rest of reported results reflecting this bad conditioning.

1. Test 2: Use of different detrending approaches

In general, there are two ways widely used to detrending a time series. The simplest method to detrend a time series is by differencing. This approach works well for data with a linear trend. If the trend is quadratic (the change in the trend also increases or decreases), then a difference of the already-differenced dataset can be taken, i.e. a second level of differencing. The second method to detrend a time series is to use a model fitting and then remove it from the original time series. This latter methodology was the chosen one to detrend the time series studied in this work.

In order to study the effect of this detrending on the final result, we also checked the detrending based on differencing. We observe that the first level of differencing approach does not work well, i.e. we do not reject completely the trend of the time series. This means that the trend is (at least) quadratic. In order to obtain a stationary time series using differencing method, we apply the second level of differencing. After this procedure, we obtain two well-defined stationary time series. This means that the trend is quadratic and this is the reason why we use a quadratic fit as optimum trend to detrend the time series presented in the paper. Fig E shows the two analyzed time series after detrending and Table C summarizes the values of transfer entropy calculated from them. In the Fig E, we can observe the different periodicities of the detrended time series compared to the previous detrending presented in the paper (Fig 2).

The values (Table C) calculated using as optimal parameters *S* and *k* those presented in the Table 1 of the main text (*S*=4, *k_SAA_*=26 and *k_GSL_*=13), are very similar to those obtained with the model fitting detrending presented in the Table 2 of the main text. The transfer information is greater from SAA to GSL time series than vice versa and the values of the TE are around 0.01 bits for the TE from SAA to GSL, the same result reported in Table 2. The statistical significance is around 90% in all cases. The values of TE from GSL to SAA are also similar (0.03-0.04 bits) and the statistical significant is little meaningful (<70%).

1. Test 3: Use of unsmooth GSL time series

If we consider the unsmooth GSL time series and apply the model fitting technique using a quadratic polynomial to detrend the time series (Fig F), we can clearly observe that we have mathematical artifacts at the earlier times (before 1850) in the GSL time series. This is likely due to that the tide-gauge records used to extend the record backwards from 1850 belong to three long discontinuous records as was pointed out by [2], being the error of the reconstruction higher in this epoch.

Anyway, we have carried out a test by using both time series without applying the penalized cubic splines to smooth them (Fig F). The transfer entropy results are shown in the Table D. The outcomes show that the transfer information flow from SAA to GSL is greater than from GSL to SAA in two of the three case studies, with statistical significance around 70-80%. In the third case, the TE value is practically the same in both senses.

In spite of these outcomes, we think that the problems associated to the input GSL time series before 1850 could be conditioning our findings. For this reason, we have carried out a new test considering only data from 1850. Due to the different spanned time, we have recalculated the optimal parameters (*S* and *k*) for both time series following the methodology proposed in the manuscript, and determined the transfer entropy. In this case, *S*=4, *k_SAA_*=19 and *k_GSL_*=6. The transfer entropy obtained from SAA to GSL is 0.271 bits and from GSL to SAA is 0.054 bits. In this case, the SAA time series is the same for the three different historical geomagnetic field models (see Fig 1a in the main text). The statistical significances are of 54% and 96%, respectively. As we can observe again, the transfer entropy from SAA to GSL is greater than from GSL to SAA. However, the statistical significance is not so high in this case. We think that, unfortunately, the number of data from 1850 can be not enough to perform an accurate analysis of transfer entropy. Nevertheless, it is interesting that we obtain the same prevalent sense of the transfer information in all these proofs, i.e. from SAA to GSL.

Finally, in order to facilitate the understanding of the results given by the TE technique, we propose a number of case studies using time series that present (and do not present) known phenomenological correlation (Fig G).

Case studies with known correlation:

We have chosen two examples of a delay between two correlated signals.

1. The time series are taken from two different sensors where the signal from Sensor 2 arrives at an earlier time than the signal from Sensor 1 (Fig Ga). Data and analysis are developed by using Matlab R2017b and R-project software. Following the same methodology explained in the main text of the manuscript, with *S*=4 and *k*=1 for both sensors, we obtain that the TE from Sensor 1 to Sensor 2 is 0.0048 bits with about 48% of significance, and from Sensor 2 to Sensor 1 is 0.037 bits with about 83% of significance. This means that knowing the Sensor 2 signal adds predictability to Sensor 1 signal, as we expected.
2. We generate two time series following the expression $x_{i}\left( t \right)=0.5x_{i}\left( t-1 \right)+\epsilon_{i}(t)$, where *i*=1,2 refers to one of the two series, *t* refers to time and $\epsilon$ is a random Gaussian distribution with mean equal to zero and standard deviation equal to 0.45. We select the same time that the original series of SAA and GSL, from 1700 to 2000 AD, and introduce a delay in the x_2_ series of 100 years (Fig Gb). We select *S*=7 and *k*=1 and calculate the TE. The result of TE from x_1_ to x_2_ is 0.39 bits with about 98% of significance and 0.35 bits from x_2_ to x_1_ with about 94% of significance. This means that x_1_ adds more predictability to x_2_ than vice versa, as we expected again.

Case study without correlation:

Two random time series (without phenomenological correlation) have been created in order to show the absence of significant TE between them. We have chosen two random homogeneous distribution with amplitude given by the optimal number of bins (*S*=4) and with the same size than the original time series (i.e. SAA and GSL time series) (Fig Gc). Its correlation coefficient is equal to 0.0157. This test not only checks the well working of the TE methodology but also is an additional proof of the significance of our results. If the TE of two random series with the same discretization (*S*=4) and sample size of the studied time series is lesser than the TE value registered in our work (Table 2 in the main text), then we have a new proof that our findings are significant. In this case, *k*=1. The TE between the first and the second series is 0.087 bits with about 34% of significance, while from second to first series the TE is 0.082 bits with about 28% of significance. These results mean that the TE between two random series is not significant, as we expected, and is lesser than the values of the TE from SAA to GSL time series (around 0.10 bits, see Table 2 in the main text), by reinforcing our outcomes.

## Supplementary Figures


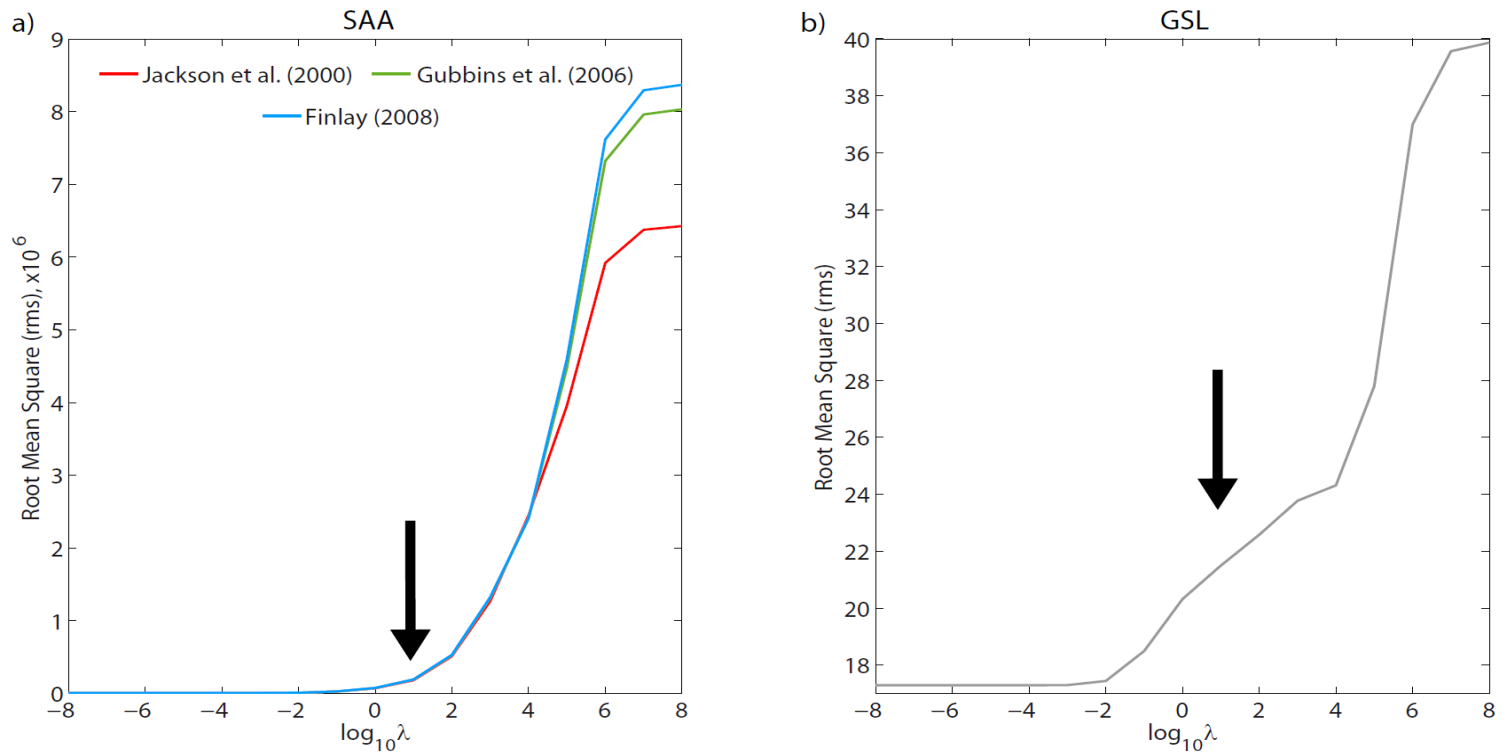


**Fig A.** Root mean square (rms) error in function on different damping parameters (*λ*). The arrows indicate the points where this parameter is optimal, according to the trade-off curves.


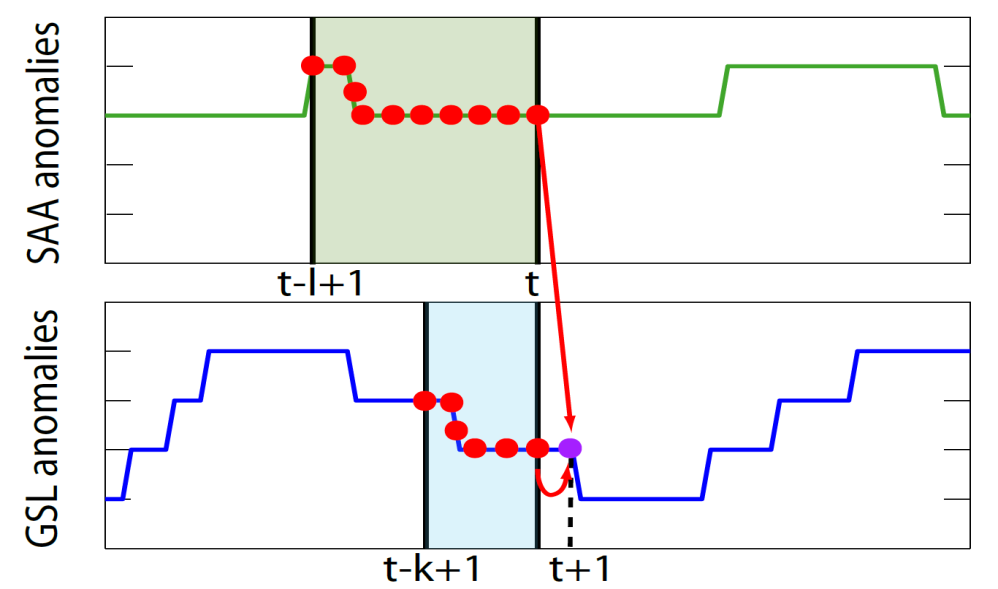


**Fig B.** Transfer entropy (red arrow) between a source *J* (SAA anomalies) and a target process *I* (GSL anomalies). Green and blue boxes indicate past states (memory) of both processes, the purple circle indicates the future value of *I* (GSL anomalies).


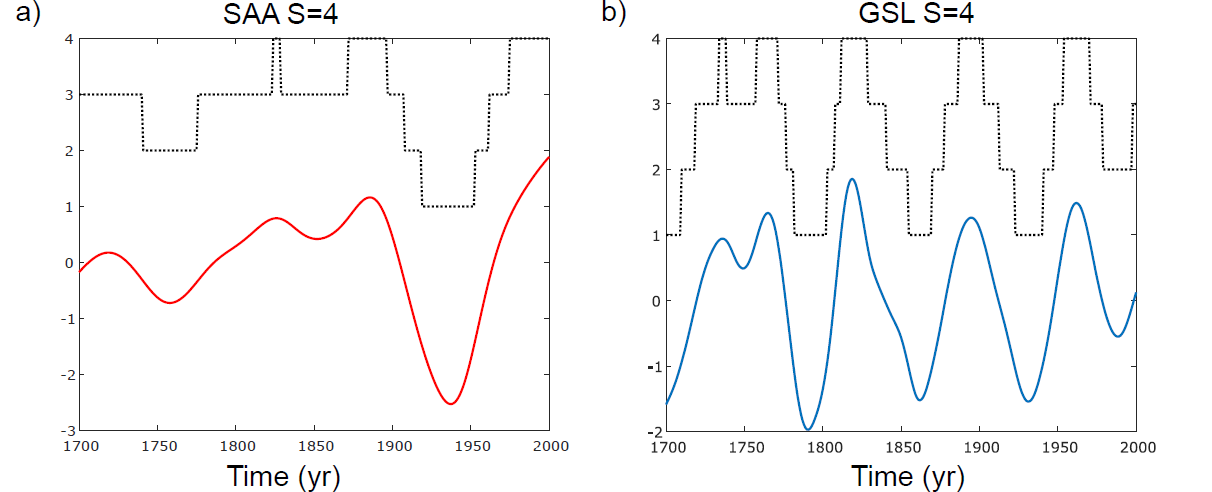


**Fig C.** Discretization of time series according to *S=*4 a) for SAA anomalies computed from [3] and b) for GSL anomalies.


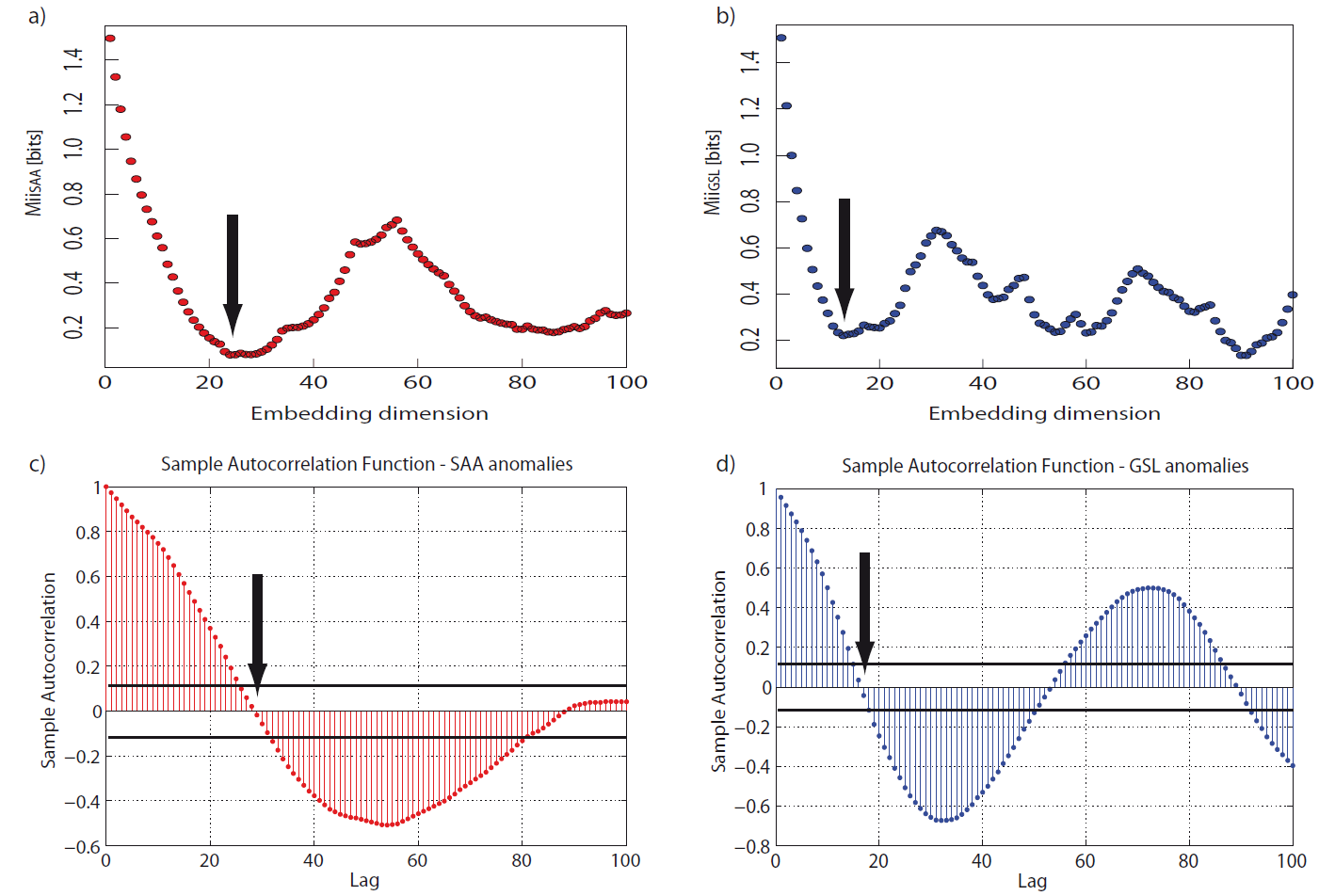


**Fig D.** Mutual information a) for SAA anomalies computed from [3] and b) for GSL anomalies. The arrows indicate the first minimum, which represents the optimal embedding dimension *k*. The sample autocorrelation function is also plotted in c) for SAA anomalies from [3] and d) for GSL anomalies. Again, the arrows mark the optimal embedding dimension given by the first zero of the autocorrelation function.


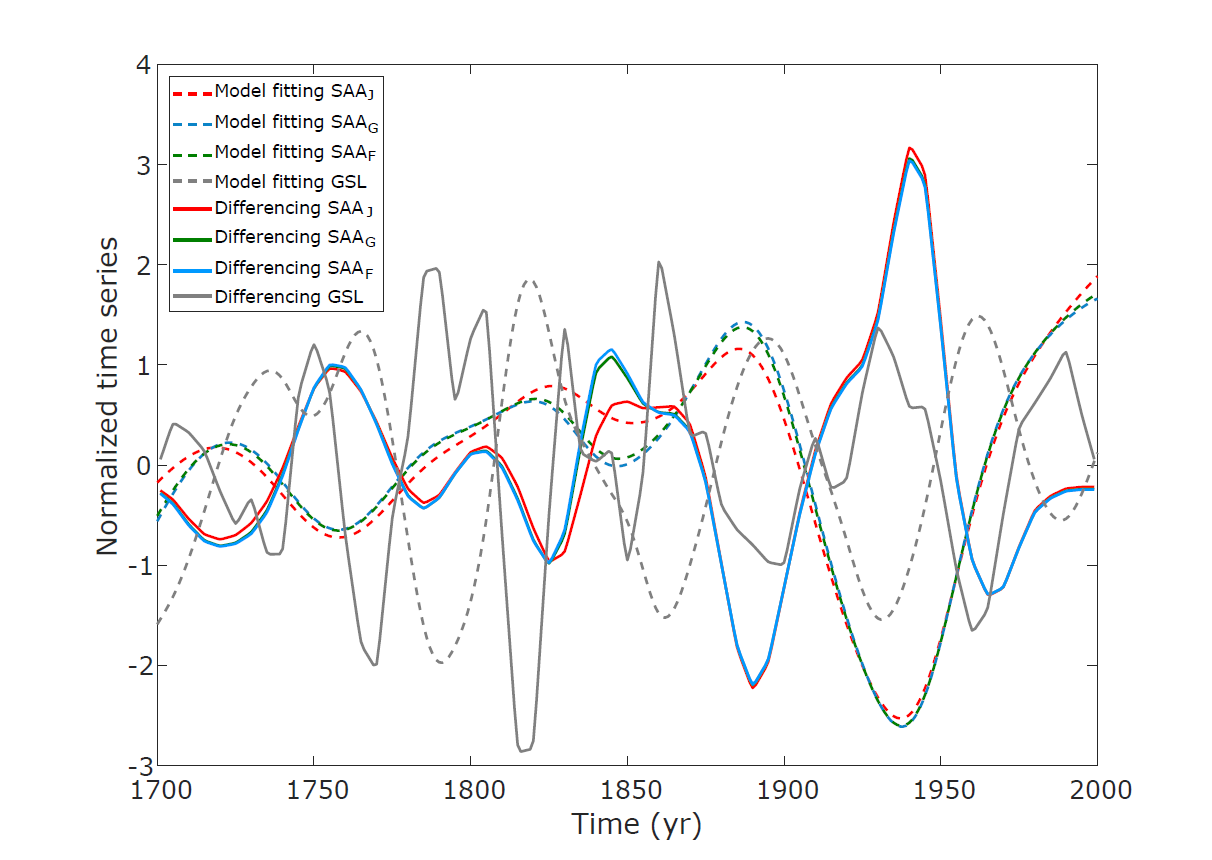


**Fig E. Evolution of the time series anomalies: test 2.** Comparison between model fitting detrending (dashed lines) and differencing detrending (solid lines). Red, green, blue lines correspond to SAA anomalies derived from [3], [4] and [5], respectively. Grey line represents the GSL anomalies. See text for further details. Time series have been normalized to zero mean and unit variance.


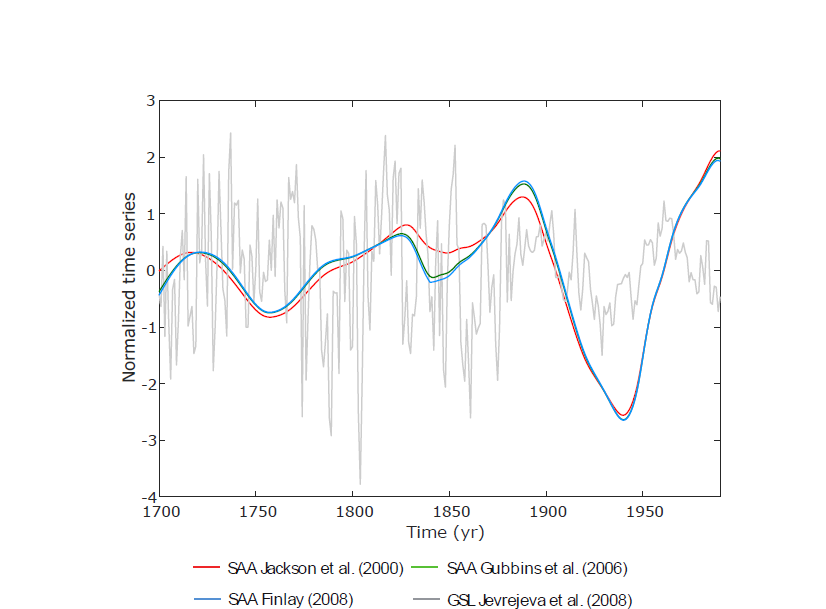


**Fig F. Evolution of the time series anomalies: test 3.** Red, green, blue lines correspond to SAA anomalies derived from [3], [4] and [5], respectively. Grey line represents the GSL anomalies. See text for further details. Both time series have been normalized to zero mean and unit variance.


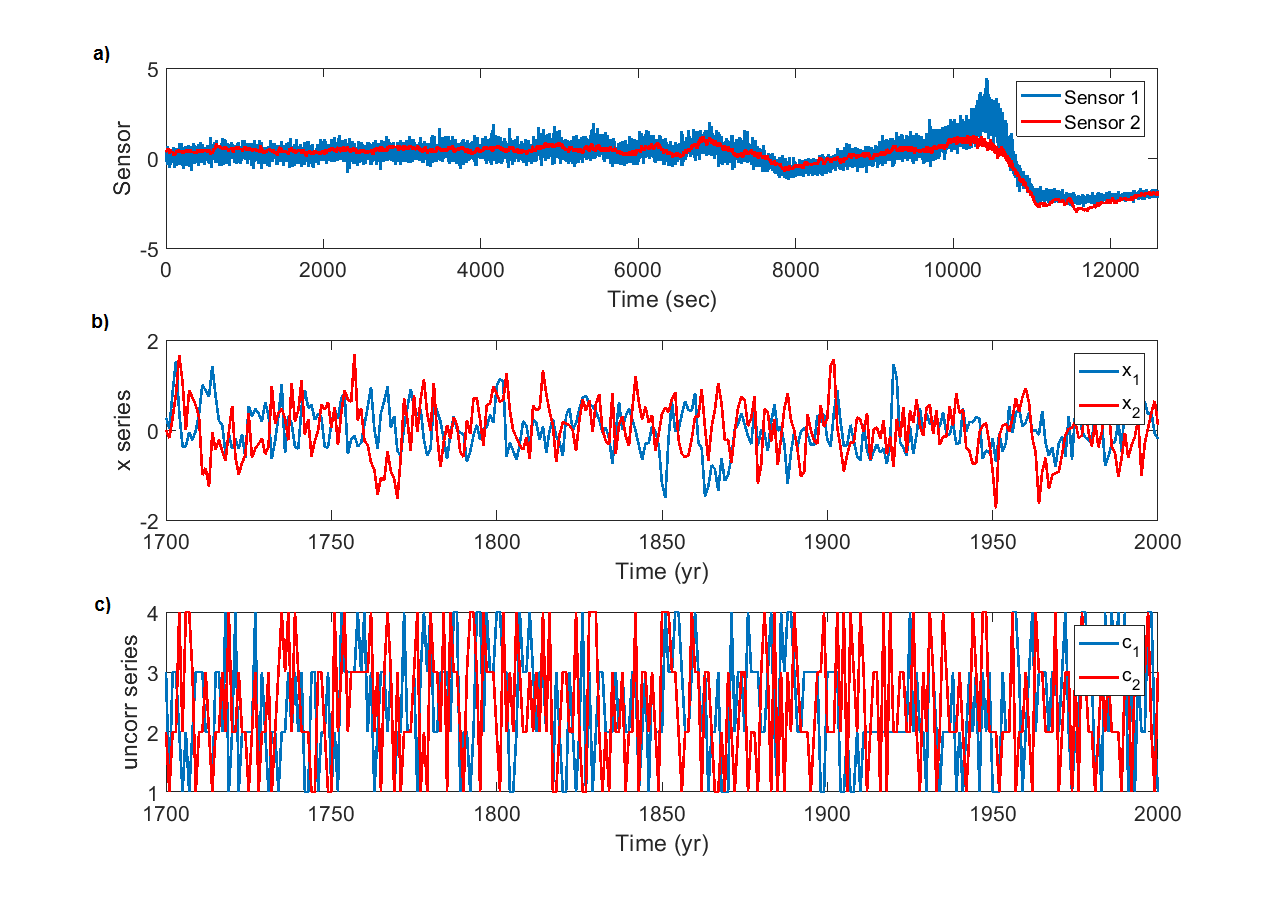


**Fig G. Evolution of the time series of the different case studies.** a) Case study 1, b) case study 2 and c) case study 3. See text for further information.

## Supplementary Tables

**Table A. Different sets of optimization parameters (*S, k*): test 1.** Different sets of optimization parameters: number of bins *S* and embedding dimension *k*, for SAA and GSL anomalies. As it can be observed, the highest values of embedding dimension are reported when *S*=3. This means that the memory of system is longer, which can be due to there is less information when the number of possible states of the system is small.

a)

| **OPTIMIZATION PARAMETERS** | | | | | | | |
| --- | --- | --- | --- | --- | --- | --- | --- |
|  | | **SAA surface extent** | | | | | **GSL** |
|  |  | *Jackson et al.* [2000] | *Gubbins et al.* [2006] | | *Finlay* [2008] | |  |
| **S** | | 5 | 5 | | 5 | | 5 |
| **k** | 23 | | | 22 | 21 | 15 | |

b)

| **OPTIMIZATION PARAMETERS** | | | | | | | |
| --- | --- | --- | --- | --- | --- | --- | --- |
|  | | **SAA surface extent** | | | | | **GSL** |
|  |  | *Jackson et al.* [2000] | *Gubbins et al.* [2006] | | *Finlay* [2008] | |  |
| **S** | | 4 | 4 | | 4 | | 4 |
| **k** | 24 | | | 26 | 26 | 13 | |

c)

| **OPTIMIZATION PARAMETERS** | | | | | | | |
| --- | --- | --- | --- | --- | --- | --- | --- |
|  | | **SAA surface extent** | | | | | **GSL** |
|  |  | *Jackson et al.* [2000] | *Gubbins et al.* [2006] | | *Finlay* [2008] | |  |
| **S** | | 3 | 3 | | 3 | | 3 |
| **k** | 35 | | | 30 | 30 | 16 | |

d)

| **OPTIMIZATION PARAMETERS** | | | | | | | |
| --- | --- | --- | --- | --- | --- | --- | --- |
|  | | **SAA surface extent** | | | | | **GSL** |
|  |  | *Jackson et al.* [2000] | *Gubbins et al.* [2006] | | *Finlay* [2008] | |  |
| **S** | | 5 | 9 | | 9 | | 11 |
| **k** | 23 | | | 26 | 23 | 13 | |

**Table B. Transfer entropy using different sets of optimization parameters (*S, k*): test 1.** Transfer entropy and statistical significance (in brackets) from SAA to GSL anomalies and from GSL to SAA anomalies. The different tables represent the values of TE with different sets of optimal number of bins *S* and optimization embedding parameters (see Table A). In bold the TE calculated with *l*=1 and optimal *k* is shown, in bold and italics when *k*=*l* and only in italics when *k* and *l* are the embedding dimension optimized for each time series (*k*=*k_SAA_* and *l*=*k_GSL_* for TE_GSL->SAA_; *k*=*k_GSL_* and *l*=*k_SAA_* for TE_SAA->GSL_).

| a) | *Jackson et al.* [2000] | *Gubbins et al.* [2006] | *Finlay* [2008] |
| --- | --- | --- | --- |
| TE_SAA->GSL_ [bits] | **0.076 (13%)**  ***0.13 (26%)***  *0.14 (11%)* | **0.090 (53%)**  ***0.12 (3%)***  *0.14 (12%)* | **0.083 (36%)**  ***0.12 (3%)***  *0.13 (4%)* |
| TE_GSL->SAA_ [bits] | **0.045 (90%)**  ***0.082 (4%)***  *0.082 (27%)* | **0.049 (94%)**  ***0.11 (13%)***  *0.11 (50%)* | **0.056 (98%)**  ***0.12 (21%)***  *0.11 (36%)* |
| With parameters given in Table Aa | | | |

| b) | *Jackson et al.* [2000] | *Gubbins et al.* [2006] | *Finlay* [2008] |
| --- | --- | --- | --- |
| TE_SAA->GSL_ [bits] | **0.091 (85%)**  ***0.15 (6%)***  *0.20 (15%)* | **0.10 (98%)**  ***0.18 (39%)***  *0.21 (21%)* | **0.11 (99%)**  ***0.18 (43%)***  *0.20 (9%)* |
| TE_GSL->SAA_ [bits] | **0.039 (67%)**  ***0.13 (24%)***  *0.12 (77%)* | **0.027 (48%)**  ***0.12 (13%)***  *0.093 (31%)* | **0.027 (48%)**  ***0.12 (15%)***  *0.091 (26%)* |
| With parameters given in Table Ab | | | |

| c) | *Jackson et al.* [2000] | *Gubbins et al.* [2006] | *Finlay* [2008] |
| --- | --- | --- | --- |
| TE_SAA->GSL_ [bits] | **0.038 (35%)**  ***0.084 (1%)***  *0.13 (0%)* | **0.043 (68%)**  ***0.12 (19%)***  *0.14 (1%)* | **0.042 (63%)**  ***0.12 (20%)***  *0.14 (1%)* |
| TE_GSL->SAA_ [bits] | **0.019 (69%)**  ***0.076 (3%)***  *0.068 (58%)* | **0.016 (48%)**  ***0.097 (24%)***  *0.075 (41%)* | **0.018 (55%)**  ***0.098 (20%)***  *0.075 (39%)* |
| With parameters given in Table Ac | | | |

| d) | *Jackson et al.* [2000] | *Gubbins et al.* [2006] | *Finlay* [2008] |
| --- | --- | --- | --- |
| TE_SAA->GSL_ [bits] | **0.021 (46%)**  ***0.021 (1%)***  0.032 (3%) | **0.021 (27%)**  ***0.030 (1%)***  0.044 (2%) | **0.021 (30%)**  ***0.025 (2%)***  0.043 (6%) |
| TE_GSL->SAA_ [bits] | **0.045 (59%)**  ***0.082 (0%)***  *0.082 (7%)* | **0.026 (32%)**  ***0.061 (0%)***  *0.061 (4%)* | **0.063 (83%)**  ***0.092 (0%)***  *0.092 (4%)* |
| With parameters given in Table Ad | | | |

**Table C. Results of transfer entropy analysis: test 2.** Transfer entropy and statistical significance (in brackets) from SAA to GSL times series and from GSL to SAA time series, with the optimal parameters (*S* and *k*) reported in the S1 File, and *l*=1.

|  | *Jackson et al.* [2000] | *Gubbins et al.* [2006] | *Finlay* [2008] |
| --- | --- | --- | --- |
| TE_SAA→GSL_ [bits] | 0.099 (90%) | 0.099 (90%) | 0.099 (87%) |
| TE_GSL→SAA_ [bits] | 0.031 (35%) | 0.041 (67%) | 0.041 (67%) |

**Table D. Results of transfer entropy analysis: test 3.** Transfer entropy and statistical significance (in brackets) from SAA to GSL anomalies and from GSL to SAA anomalies, with the optimal parameters (*S* and *k*) reported in the S1 File, and *l*=1.

|  | *Jackson et al.* [2000] | *Gubbins et al.* [2006] | *Finlay* [2008] |
| --- | --- | --- | --- |
| TE_SAA→GSL_ [bits] | 0.032 (83%) | 0.027 (68%) | 0.029 (77%) |
| TE_GSL→SAA_ [bits] | 0.018 (19%) | 0.028 (72%) | 0.023 (55%) |

**References**

[1] Marschinski R, Kantz H. Analysing the information flow between financial time series: An improved estimator for transfer entropy. Eur. Phys. J. B. 2002; 30: 275–281. doi: 10.1140/epjb/e2002-00379-2.

[2] Jevrejeva S, Moore JC, Grinsted A, Woodworth PL. Recent global sea level acceleration started over 200 years ago? Geophys. Res. Lett. 2008; 35: L08715. doi:10.1029/2008GL033611.

[3] Jackson A, Jonkers ART, Walker MR. Four centuries of geomagnetic secular variation from historical records. Philos. Trans. R. Soc. Lond. A. 2000; 358 (1768): 957-990.

[4] Gubbins D, Jones AL, Finlay CC. Fall in Earth’s Magnetic Field is erratic. Science. 2006; 312 (5775): 900-902.

[5] Finlay CC. Historical variation of the geomagnetic axial dipole. Phys. Earth Planet. Interiors. 2008; 170: 1-14.
